# Case Report: The great mimicker: cerebral syphilitic gumma

**DOI:** 10.3389/fmed.2025.1600412

**Published:** 2025-06-24

**Authors:** Jiahui Liu, Kun Sun, Yanxia Deng, Lianshu Ding

**Affiliations:** Department of Neurosurgery, The Affiliated Huaian No.1 People's Hospital of Nanjing Medical University, Nanjing Medical University, Huai'an, Jiangsu, China

**Keywords:** cerebral syphilitic gumma, neurosyphilis, cerebral syphilis, case report, neurosurgery, infectious disease

## Abstract

Cerebral syphilitic gumma, a rare form of neurosyphilis, poses significant diagnostic challenges due to its imaging similarities to gliomas and brain metastases. This report details the diagnostic and therapeutic journey of a patient with cerebral syphilitic gumma admitted to the Department of Neurosurgery, Nanjing Medical University Affiliated Huaian First Hospital in February 2025. Initially misdiagnosed with a brain malignancy, the accurate diagnosis was established intraoperatively and confirmed postoperatively through histopathological examination. This case highlights the importance of considering neurosyphilis in differential diagnoses of intracranial lesions, especially when conventional imaging and clinical presentations are inconclusive.

## Introduction

In recent years, syphilis has re-emerged as a global public health concern, with increasing incidence rates in high-income countries (1). Neurosyphilis, which can develop days after *Treponema pallidum* infection, is categorized into early and late stages. Early neurosyphilis typically presents as asymptomatic or with meningitis, while late neurosyphilis manifests as progressive dementia and tabes dorsalis (2). Cerebral syphilitic gumma, or cerebral gumma, constitutes 0.1–0.6% of intracranial tumors. Typically, diagnosis is relatively straightforward as patients often present with skin, eye, or ear symptoms prior to confirmation. However, misdiagnosis becomes highly probable when the initial symptoms are non-specific neurological manifestations, particularly in cases of coma where obtaining a detailed medical history is challenging. Neurosyphilis frequently exhibits ischemic infarction and demyelination changes on neuroimaging studies (3). Intracranial syphilitic gumma, often mimicking high-grade gliomas and metastases, poses significant diagnostic challenges. This report presents a case of cerebral syphilitic gumma initially manifesting as seizures and somnolence, which was misdiagnosed as a malignant brain tumor. Our aim is to provide valuable clinical guidance and insights for neurosurgeons in managing similar cases.

## Case data

**Figure 1:**
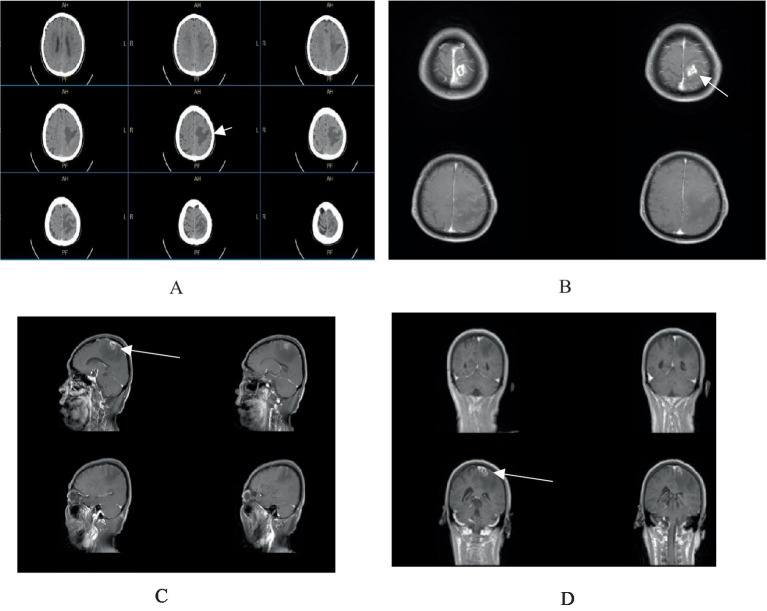
**(A)** Cranial CT scan reveals an irregular hypodense lesion in the left parietal lobe. **(B–D)** Contrast-enhanced cranial MRI demonstrates a ring-enhancing lesion in the left parietal lobe, measuring approximately 23 × 17 mm, surrounded by extensive non-enhancing perilesional edema, with irregular morphology of the adjacent meninges.

A 64-year-old male from Huaian, Jiangsu, was admitted in February 2025 with a one-week history of right-sided limb weakness and a seizure episode. He had no significant medical, surgical, family, or allergy history. Initially diagnosed with cerebral infarction at a local hospital, conservative management proved ineffective. On admission, he presented with agitation and a Glasgow Coma Scale score indicative of sedative coma. Physical examination revealed equal and reactive pupils, no neck stiffness, and right-sided limb paralysis. Cranial magnetic resonance imaging (Figure 1) showed a 23 × 17 mm ring-enhancing lesion in the left parietal lobe with surrounding edema and irregular adjacent meninges, suggesting a possible glioma or metastasis.

## Treatment and diagnosis

After emergency preoperative assessments and symptomatic management, including dehydration, seizure prophylaxis, sedation, and fluid resuscitation, the patient underwent additional testing. Tumor markers and PET-CT were performed to rule out brain metastasis. Preoperative serological tests revealed HIV-negative but positive *Treponema pallidum* antibodies, prompting further TPPA (positive) and RPR (titer 1:4) tests. A dermatology consultation established a diagnosis of latent syphilis, and the patient was treated with intramuscular benzathine penicillin (2.4 million units).

Despite these measures, the patient still had headaches and right-sided hemiplegia. Given the ongoing suspicion of a glioma, a craniotomy was performed. Intraoperatively, inflammatory granulation tissue along the veins and a hard glial hyperplasia band with necrotic colloid material were observed in the left parietal lobe, atypical for glioma.

**Figure 2:**
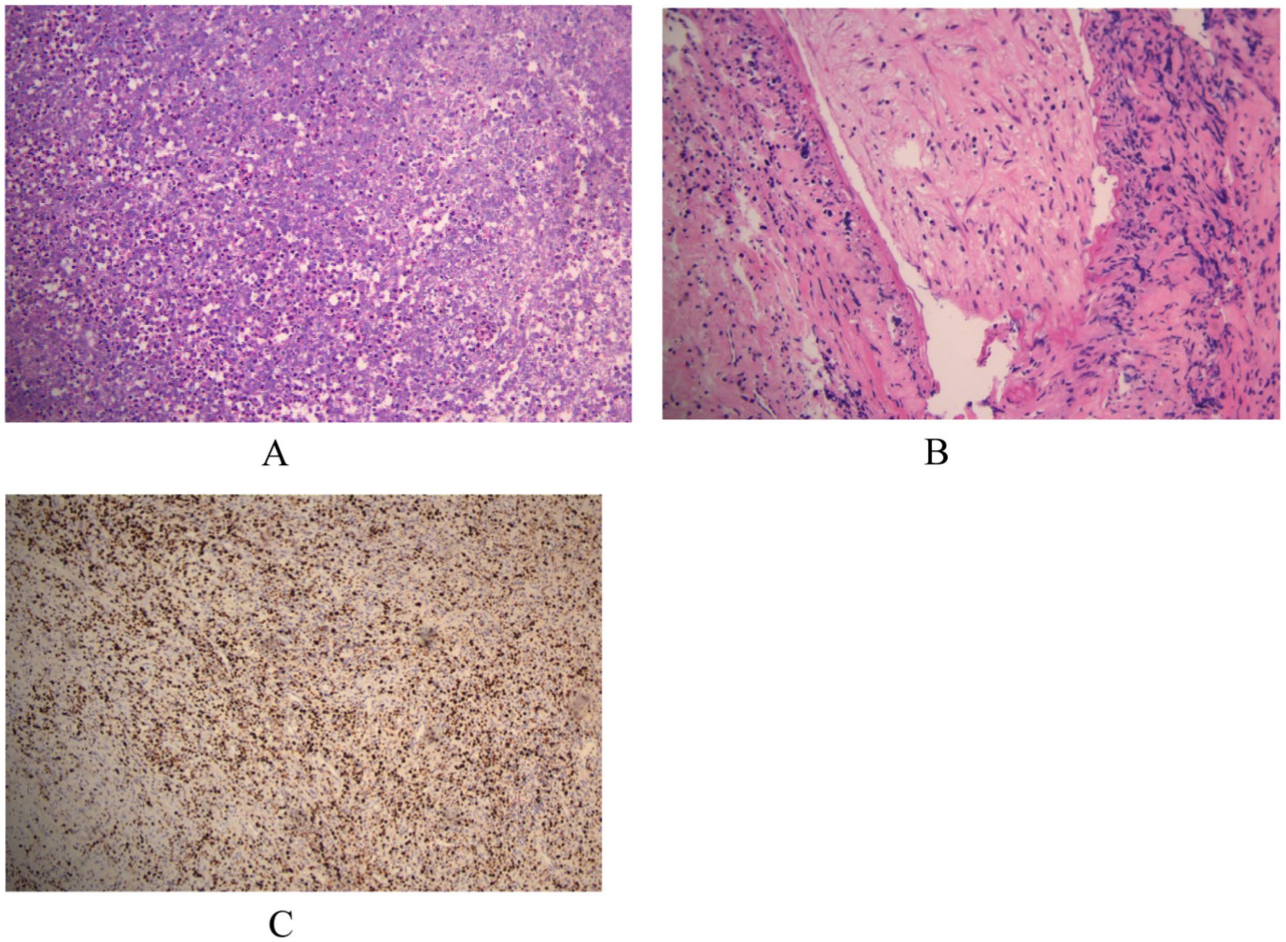
**(A,B)** Microscopic examination reveals infiltration by chronic inflammatory cells, plasma cells, and eosinophils, with focal areas of obliterative endovasculitis. **(C)** Immunohistochemical staining with horseradish peroxidase-diaminobenzidine (HRP-DAB) shows the following profile: CD20(+), CD68(+), CD79α(+), CD163(+), CD3(−), CD5(−), CD1α(−), CK(−), GFAP(−), Ki-67 (50%+).

Postoperative histopathological analysis (Figure 2) showed chronic inflammatory cell infiltration, plasma cells, eosinophils, and foci of obliterative endovasculitis. Immunohistochemistry revealed CD20(+), CD68(+), CD79α(+), CD163(+), and Ki-67 (50%+), with CD3(−), CD5(−), CD1α(−), CK(−), and GFAP(−). To further confirm neurosyphilis, postoperative lumbar puncture was performed. CSF examination showed: clear appearance, total cell count 45 × 10^6^/L, protein 0.5 g/L, glucose 2.5 mmol/L, chloride 122.2 mmol/L, TPPA positive, RPR titer 1:2. Combining serology, CSF, intraoperative findings, postoperative pathology, and immunohistochemistry, the patient was diagnosed with the rare cerebral syphilitic gumma.

At discharge, the patient had normal limb strength and no obvious headache. To ensure the best medical care, the patient was transferred to a specialized infectious disease hospital for further neurosyphilis treatment. During follow-up, the patient reported no headache, limb weakness, or seizures, and neurological examination was normal. Serological tests showed positive *Treponema pallidum* antibodies but otherwise normal results. Follow-up CSF examination revealed: clear appearance, total cell count 2 × 10^6^/L, protein 0.2 g/L, glucose 2.3 mmol/L, chloride 125.2 mmol/L, and TPPA negative.

## Discussion

Neurosyphilis can develop at any stage of syphilis, most frequently in its tertiary form, and was predominantly observed in the pre-antibiotic era. It is relatively rare today (4). Latent syphilis, which is asymptomatic, remains infectious and is often detected incidentally. Approximately 30% of untreated latent syphilis cases may advance to late-stage syphilis (5). Symptomatic neurosyphilis can clinically resemble dementia, cerebral infarction, meningitis, and brain tumors, with symptoms such as seizures, headaches, vomiting, papilledema, hemiplegia, cranial nerve involvement, and cognitive impairment. Cerebral syphilitic gumma, a type of neurosyphilis, mimics gliomas and meningiomas in imaging and is clinically similar to gliomas, making it a significant diagnostic challenge for clinicians (3, 6, 7).

In our case, cerebral syphilitic gumma mimicked cerebral infarction. The patient presented with limb weakness, and the local hospital misdiagnosed the left parietal hypodense lesion on cranial CT as a cerebral infarction, resulting in ineffective treatment. Upon the patient’s arrival at our hospital, we revised the diagnosis. After comprehensive examination, we considered glioma, as cerebral syphilitic gumma closely resembles glioma both clinically and radiologically. High-grade gliomas show contrast enhancement on T1-weighted MRI sequences, high-signal brain edema on T2-weighted/fluid-attenuated inversion recovery (T2/FLAIR), and irregular ring-enhancement with central necrosis and peritumoral edema on enhanced MRI (8). Cerebral syphilitic gumma also presents with T1 hyperintensity, high-signal brain edema on T2/FLAIR, and central necrosis on enhanced MRI (6). Notably, both cerebral syphilitic gumma and high-grade gliomas show increased metabolism of 18F-2-fluoro-2-deoxy-D-glucose (FDG) on PET-CT, further complicating the diagnostic distinction (9).

Compared to previous neurosyphilis case reports (10–12), this case is unique in the following ways. First, the patient’s onset age was 64, older than those with similar syphilitic gummas. Clinically, he presented with limb weakness and seizures, which are common in most neurosyphilis cases. However, the patient was somnolent and uncooperative upon admission, masking some typical neurological symptoms and increasing diagnostic difficulty. Second, regarding radiological features, the cerebral gumma in this case showed as a solitary left parietal ring-enhancing lesion with surrounding edema on MRI. This differs from recent reports that mainly describe large patchy abnormal signals, multiple ring-enhancing lesions, and adjacent meningeal enhancement. Lastly, in diagnosis and treatment, although preoperative treponemal antibodies were positive, the patient’s somnolence and quadriplegia made urgent surgical decompression necessary to prevent functional deterioration, a situation unreported in prior studies.

This case uniquely highlights the diagnostic and therapeutic challenges, atypical imaging features, and management issues in neurosyphilis. The primary diagnostic difficulty lay in the nonspecific clinical presentation and imaging findings of the patient. Clinicians are reminded to consider neurosyphilis in patients presenting with nonspecific neurological symptoms, particularly when routine treatments are ineffective or imaging findings are ambiguous. Second, although the cerebral gumma showed ring enhancement on MRI similar to gliomas, MRS revealed only slight decreases in Cho, Cr, and NAA levels, unlike the significant changes seen in gliomas (13). This suggests MRS could aid in differentiating between cerebral gumma and glioma. However, MRS is not widely used in clinical practice due to technical and interpretive demands. Thus, integrating advanced imaging like MRS with routine radiological exams is crucial for enhancing pre-surgical diagnostic accuracy. Lastly, when treating patients with cerebral syphilitic gumma and neurological deterioration, the choice between craniotomy for decompression and conservative anti-syphilis treatment remains unclear in previous literature. Experts in infectious diseases and neurosurgery should establish unified guidelines. We prioritize saving lives for patients with functional decline. Postoperative standardized neurosyphilis treatment and individualized plans are needed.

## Conclusion

For patients with neurosyphilis, particularly those with non-specific symptoms or unknown/hidden medical histories, integrating serological and cerebrospinal fluid tests (e.g., TPPA and TRUST) is crucial for accurate diagnosis. Notably, cerebral syphilitic gumma, a chronic granulomatous form of neurosyphilis, can be effectively differentiated using magnetic resonance spectroscopy (MRS). This technique enhances preoperative diagnostic precision and helps avoid unnecessary surgical interventions. Furthermore, appropriate syphilis treatment is indispensable and significantly impacts patient outcomes.

## Data Availability

The original contributions presented in the study are included in the article/Supplementary material, further inquiries can be directed to the corresponding author.
