# Supplementary figures and images for: Case Report: The great mimicker: cerebral syphilitic gumma
Source: Front Med (Lausanne). 2025 Jun 24;12:1600412. doi: 10.3389/fmed.2025.1600412 (PMC12234315; doi:10.3389/fmed.2025.1600412)

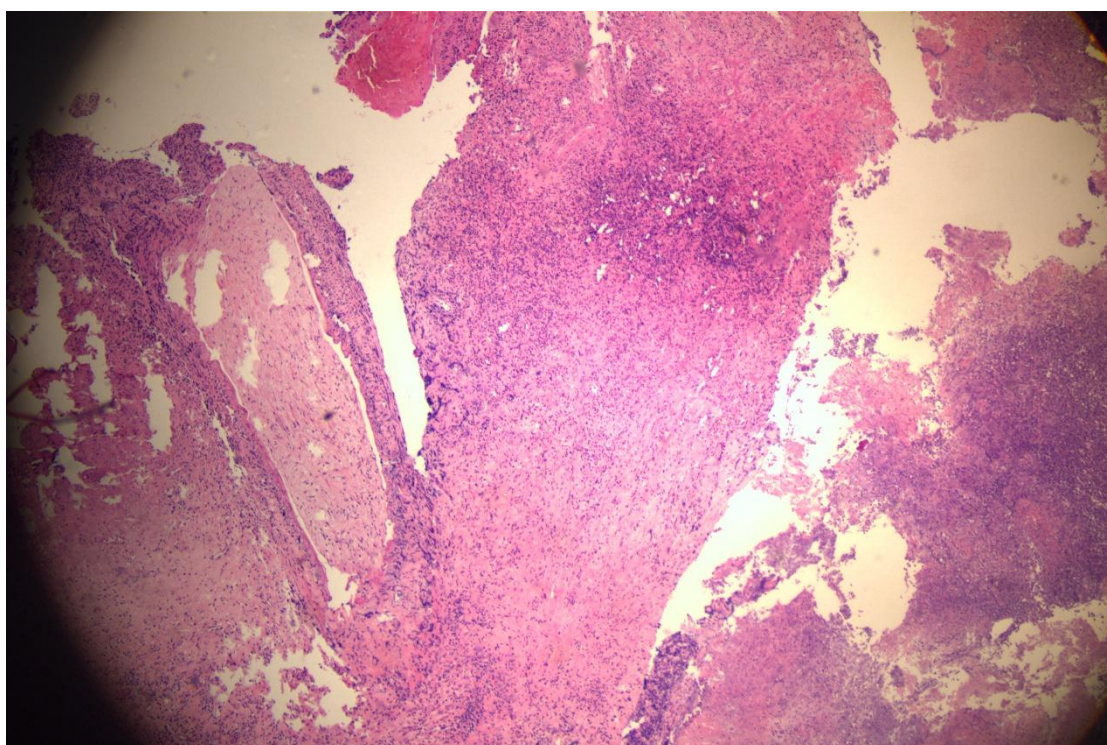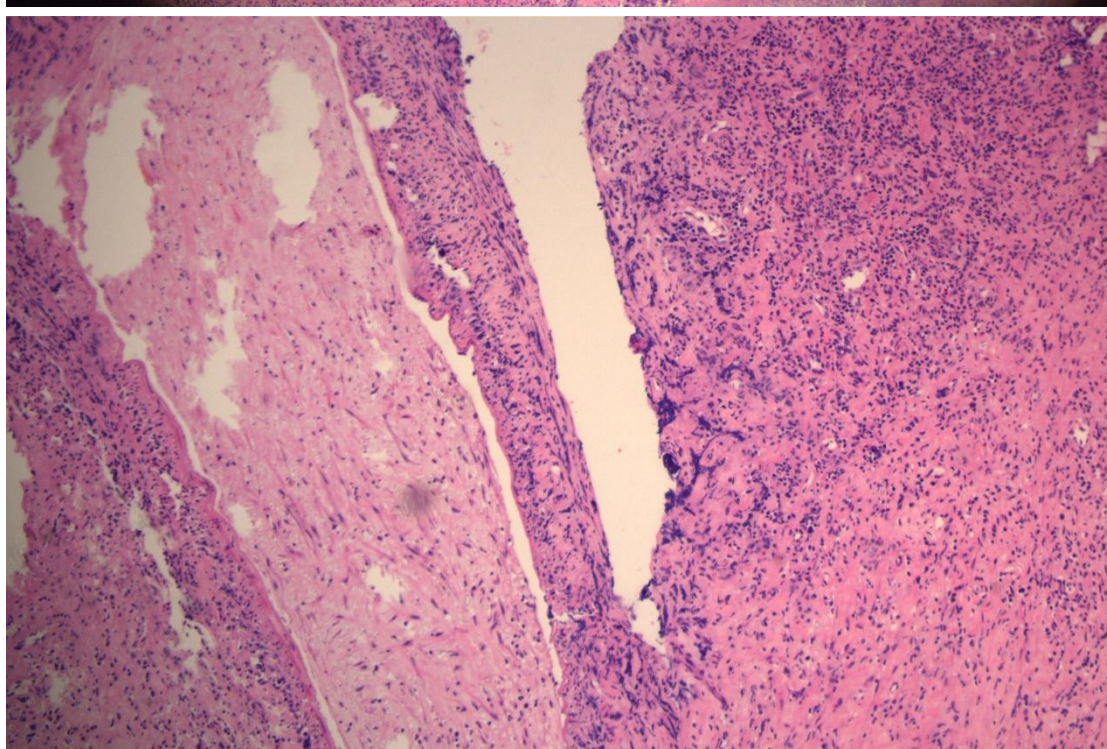

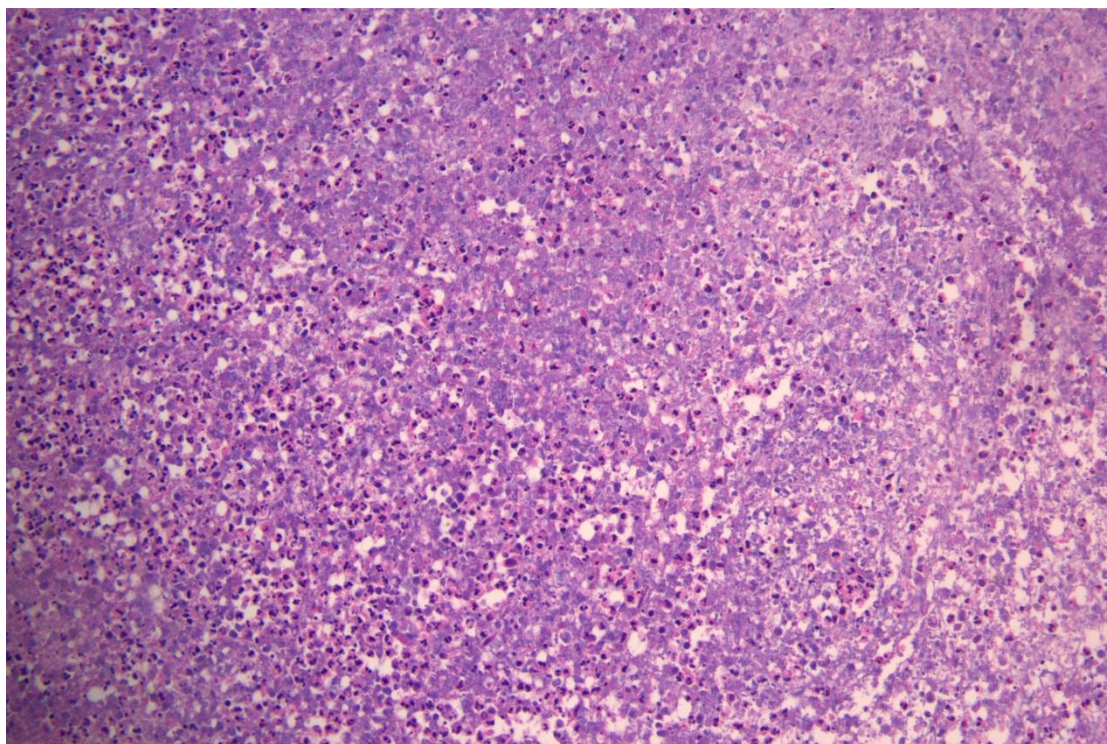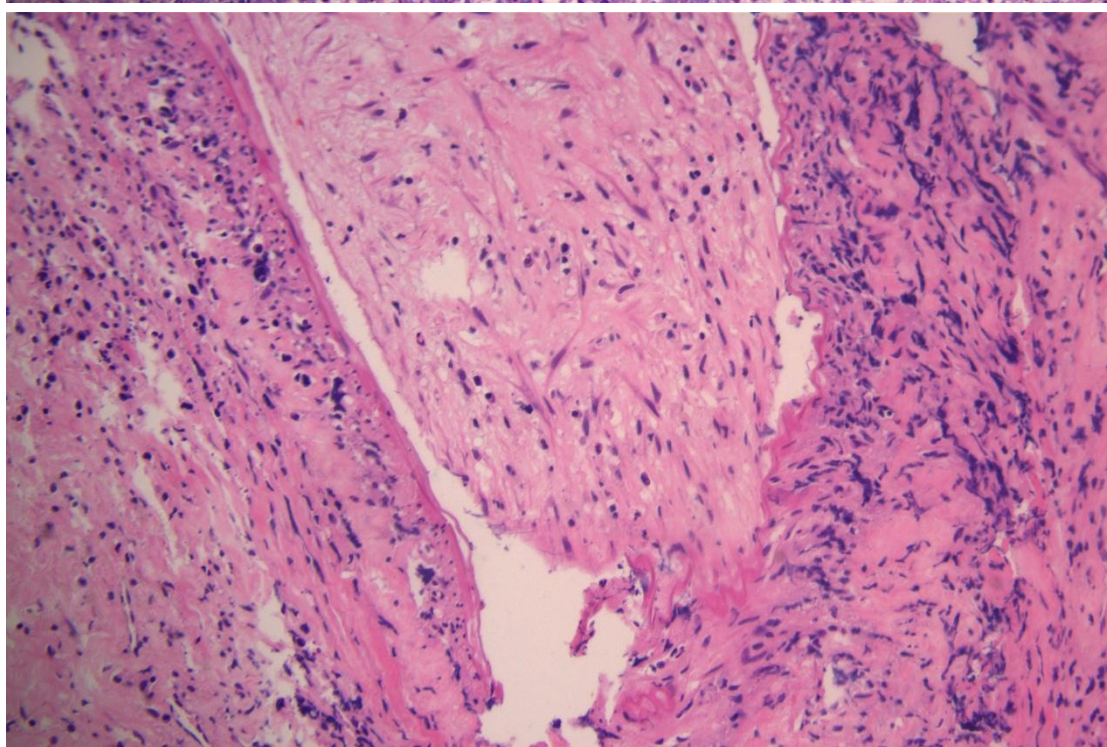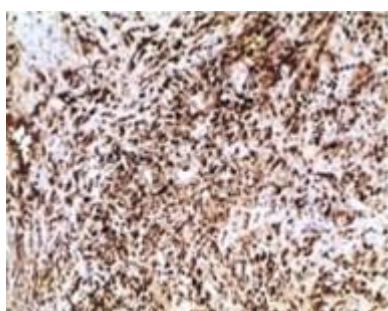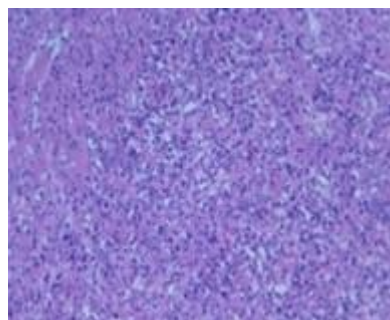

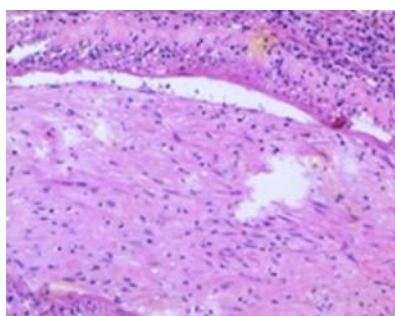

Supplement: Supplementary file 1 [file Data_Sheet_1.PDF]

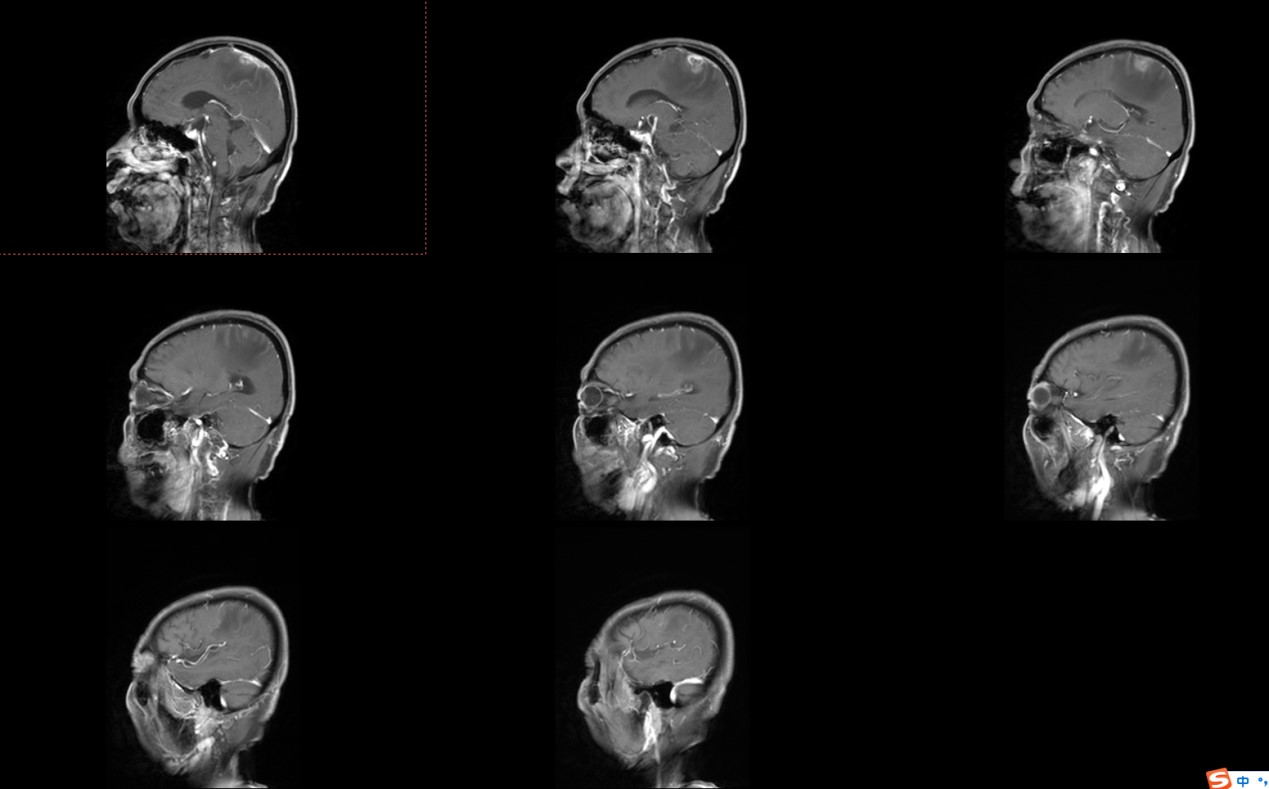

Supplement: Supplementary file 2 [file Image_1.JPEG]

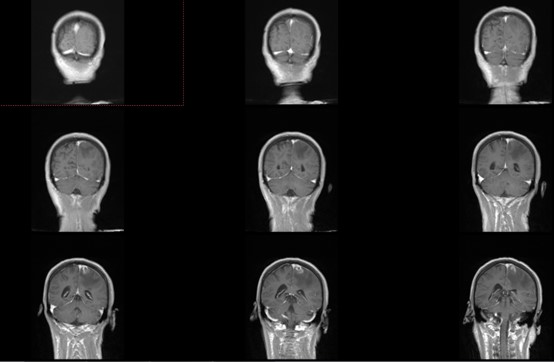

Supplement: Supplementary file 3 [file Image_2.JPEG]
